# Healthcare-seeking of medical students: the effect of socio-demographic factors, health behaviour and health status – a cross-sectional study in Hungary

**DOI:** 10.1186/s12889-023-17041-4

**Published:** 2023-10-30

**Authors:** Afriza Umami, Viktória Zsiros, Ágnes Maróti-Nagy, Zsuzsanna Máté, Sudalhar Sudalhar, Regina Molnár, Edit Paulik

**Affiliations:** 1 Department of Public Health, Albert Szent-Györgyi Medical School, University of Szeged, Dóm tér 10, Szeged, H-6720 Hungary; 2 Stikes Muhammadiyah Bojonegoro, Bojonegoro, Indonesia

**Keywords:** Healthcare-seeking, Medical students, General practitioner, Psychologist

## Abstract

**Background:**

Medical students are more likely to have various physical and psychological issues, but less information is available about the healthcare-seeking behaviour for physical and mental health issues. The aim of this study is to determine the factors affecting medical students’ healthcare-seeking when visiting a general practitioner (GP) and/or psychologist.

**Methods:**

688 medical students (326 International and 362 Hungarian,) participated in a cross-sectional study. The information was gathered using a self-administered online questionnaire and covered socio-demographic background, health behaviour, general and mental health status and healthcare-seeking. For analysing adjusted associations, multivariable logistic regression models were used.

**Results:**

Overall, 56.8% of medical students visit the GP; and 17.2%, the psychologist. Hungarian medical students visited the GP with chronic diseases, International medical students were more likely to visit a GP when they encountered sexual activity and had chronic diseases. Moreover, there was a significant correlation between sex, alcohol consumption, and perceived stress in the total sample of psychologist visits. When Hungarian medical students were in their clinical years and had a poor self-rated mental health, they were more likely to visit a psychologist. Whereas female international medical students and those who had poor self-rated mental health were more likely to seek psychological help.

**Conclusion:**

Students visit a GP and/or psychologist is associated with a variety of factors, including socio-demographic background, health behaviours, and health issues. Medical schools should encourage help-seeking behaviours and early disclosure of medical students. Their ability to grasp healthcare attitudes and designing treatments will be important for both their academic success and future profession.

## Background

The absence of seeking out physical or mental health care among medical students is acknowledged as an increasing prevalent issue by the literature [1, 2]. Medical students are reluctant to seek out treatment, instead, they show preference to seek help from informal sources, such as friends and family [3]. Informal medical care from colleagues is an element of the culture of doctors and may be an example of what future doctors learn from their role models [3, 4]. A high prevalence of mental health illnesses, lack of or delay in obtaining health care, self-diagnosis, self-prescribing and dangerous behaviours have all been observed among qualified doctors [5]. Medical students retrieve attitudes and values from professional role models, and they could also adopt behavioural traits that encourage them to get informal care for physical and mental health issues [4].

In a survey conducted at a British medical school, it has been discovered that 43% of the students had unofficially contacted a doctor who was a friend or family member, 9.2% had initiated their own investigations, and 25% had been examined by a colleague [6]. Additionally, 90% of the respondents in North American questionnaire-based research conducted in nine medical schools have reported a need for medical attention while in training. Two-thirds of them has received informal care from colleagues and half requested that a fellow student do a physical examination [7]. College students in China were found to be more reliant on seeking out online health information (OHI), just 32.4% followed up with doctors, and around 20% experienced internet hacking/fraud which will have a negative impact on their health in the future [8]. The study found that Chinese students rely on OHI to manage both their own and others’ health without sufficient knowledge/skills to identify misinformation [8].

The prevalence of mental distress among medical students ranged between 12.2 and 96.7% according to a systematic review that was conducted on 16 studies that were identified outside of North America in the English-speaking world [9]. According to a study conducted in Hungary [10], Hungarian students reported less mental well-being than students from the Mediterranean region, Israel, and Scandinavia. Furthermore, it was discovered that 49.7% of foreign students in Hungary who self-reported their mental health had poor mental health [11]. Meanwhile, the chronic disease burden is increasing globally. The study reported that 41.5% of medical students in Morocco confirmed having a chronic disease, among which 80% were under treatment [12]. Furthermore, research conducted among residents of various specialties in Saudi Arabia reported that chronic disease was reported by 29.1% [13].

Researchers should be aware of the physical and mental health issues for which students are likely to require assistance when analysing their behaviour of seeking health care [4]. Medical students seem to be more distressed than the population in general and more anxious than non-students of same ages [2, 14]. Studying as well as having concerns about competency and achievement leads to the development of stress among medical students [15]. Nonetheless, there is little information available on the physical health of medical students, their behaviour when seeking assistance for physical and mental health issues, and how it is related to the accessibility of issues or other barriers to receiving different types of care [4]. Although the prevalence of physical illness among medical students remains uncertain, there is some evidence to suggest that they will have significant health concerns during their training [16].

**Table 1:** Characteristics of respondents by Hungarian and international medical students

Characteristics	Total (688)		Hungarian (362)		International (326)		P
	N	%	N	%	N	(%)	
*Socio-demographic factors*							
Age (years)							
18–25	596	86.6	326	90.1	270	82.8	0.005
26–37	92	13.4	36	9.9	56	17.2	
Sex							
Male	268	39.0	117	32.3	151	46.3	< 0.001
Female	420	61.0	245	67.7	175	53.7	
Years of study							
Preclinical	415	60.3	191	52.8	224	68.7	< 0.001
Clinical	273	39.7	171	47.2	102	31.3	
Relationship status							
Not in a relationship	425	61.8	181	50.0	244	74.8	< 0.001
In a relationship	263	38.2	181	50.0	82	25.2	
Economic status							
Low income	247	35.9	161	44.5	86	26.4	< 0.001
High income	441	64.1	201	55.5	240	73.6	
*Health behaviours*							
Smoking							
No	533	77.5	285	78.7	248	76.1	0.405
Yes	155	22.5	77	21.3	78	23.9	
Alcohol consumption							
No	206	29.9	80	22.1	126	38.7	< 0.001
Yes	482	70.1	282	77.9	200	61.3	
Sexual intercourse							
No	324	47.1	151	41.7	173	53.1	0.003
Yes	364	52.9	211	58.3	153	46.9	
*General health status*							
Chronic disease							
No	573	83.6	294	81.2	281	86.2	0.078
Yes	113	16.4	68	18.8	45	13.8	
BMI							
≥ 25.0 kg/m^2^	155	22.5	58	16.0	97	29.8	< 0.001
≤ 24.9 kg/m^2^	533	77.5	304	84.0	229	70.2	
Self-rated general health							
Good	460	66.9	240	66.3	220	67.5	0.741
Poor	228	33.1	122	33.7	106	32.5	
*Mental health status*							
Perceived stress scale (PSS)							
Lower	545	79.2	276	76.2	269	82.5	0.043
Higher	143	20.8	86	23.8	57	17.5	
Self-rated mental health							
Good	338	49.1	174	48.1	164	50.3	0.557
Poor	350	50.9	188	51.9	162	49.7	
*Healthcare-seeking*							
Visiting a GP							
No	297	43.2	153	42.3	144	44.2	0.614
Yes	391	56.8	209	57.7	182	55.8	
Visiting a psychologist							
No	570	82.8	290	80.1	280	85.9	0.045
Yes	118	17.2	72	19.9	46	14.1	

The literature consistently recognized the high prevalence of mental distress among medical students [9, 17–19] but physical health of medical students is less understood [4, 20] however, one longitudinal study has found that the physical health can decline during medical school years [21]. Medical students often refrain from disclosing and seeking help [20–22], which could potentially endanger both themselves and their patients. There is a scarcity of literature on the factors influencing their choices regarding seeking help or disclosing health conditions [20]. Other studies tend to look at the role of the medical school in monitoring and improving student welfare that is mainly related to mental distress [23] and the importance of stress management programmes [24], rather than the role of general practitioners (GPs) in the support and management of physical and mental health. Hence, this study focusing to investigate the visiting the GP and/or the psychologist and the associated factors among medical students. Moreover, medical students experience a higher incidence of health issues compared to students in other fields [2, 14]. This can be attributed to the significant risk factor associated with pursuing education at a medical university and subsequently working as a doctor [4]. It is widely recognized that doctors often face challenges related to their mental and physical well-being. Hence, it is crucial to examine their health-seeking behaviour, as improving this aspect will be vital for their future healthcare needs.

## Methods

### Study design and participants

A cross-sectional study was employed among medical students at the University of Szeged (USZ), Hungary. The study was carried out between April and October 2021. The medical students from the first to sixth years were invited to participate in the online survey using convenience sampling. The survey was completed by 688 students in total (n = 362 Hungarian and n = 326 international students). The participation was voluntary and anonymous.

The total number of medical students enrolled at the University of Szeged was 2,382 students (Hungarian students 1,233 and international students 1,145) in the 2020/2021/2 academic year. The sample size was taken by calculating the 95% confidence level, a margin of error (ε) of 5%, and 50% population proportion.

**Table 2:** Univariable logistic regression analysis of visiting a GP. OR = odds ratio, CI = confidence interval, BMI = body mass index

Characteristics		Total (688)			Hungarian (362)			International (326)	
	OR	95% CI	p value	OR	95% CI	p value	OR	95% CI	p value
*Socio-demographic factors*									
Age (years)									
18–25			1			1			1
26–37	1.50	0.95–2.37	0.081	1.75	0.83–3.68	0.134	1.39	0.77–2.52	0.269
Sex									
Male			1			1			1
Female	1.14	0.83–1.55	0.402	1.03	0.66–1.60	0.900	1.24	0.80–1.92	0.336
Years of study									
Preclinical			1			1			1
Clinical	1.15	0.85–1.57	0.357	1.01	0.66–1.53	0.954	1.34	0.83–2.16	0.224
Relationship status									
Not in a relationship			1			1			1
In a relationship	1.17	0.86–1.61	0.301	1.02	0.67–1.55	0.915	1.42	0.85–2.37	0.180
Economic status									
Low income			1			1			1
High income	0.91	0.66–1.25	0.561	0.79	0.52–1.20	0.280	1.13	0.69–1.86	0.611
*Health behaviours*									
Smoking									
No			1			1			1
Yes	0.99	0.69–1.43	0.987	0.97	0.58–1.61	0.906	1.03	0.62–1.72	0.906
Alcohol consumption									
No			1			1			1
Yes	1.48	1.07–2.06	**0.018**	1.15	0.70–1.90	0.575	1.81	1.16–2.85	**0.010**
Sexual intercourse									
		No	1			1			1
Yes	1.50	1.11–2.04	**0.008**	1.06	0.69–1.61	0.799	2.21	1.41–3.45	**0.001**
*General health status*									
Chronic illness									
No			1			1			1
Yes	3.79	2.31–6.21	**< 0.001**	3.85	2.02–7.34	**< 0.001**	3.70	1.72–7.96	**0.001**
BMI									
≥ 25.0 kg/m^2^			1			1			1
≤ 24.9 kg/m^2^	0.69	0.47–0.99	**0.045**	0.56	0.31–1.03	0.061	0.75	0.46–1.21	0.248
Self-rated general health									
Good			1			1			1
Poor	1.18	0.86–1.64	0.293	1.13	0.73–1.77	0.564	1.24	0.77–1.98	0.363

### Medical treatment of the students at the University of Szeged

The medical services available to medical students are easily accessible and located in the city centre. There are general practitioners in primary care, where services are provided only through appointments with a GP through the modulo platform. Secondary care of all students (regardless of the type of insurance) is provided at the clinics of the Albert Szent-Györgyi Clinical Center. In each case students need to contact the institutions’ own reception desk first. If students need emergency assistance, they can come to emergency care or call the ambulance service.

In addition, there is a student counselling centre as well as a local psychologist. Medical and counselling services are available in Hungarian, English and also German, however staff available for English and German are limited. Hungarian and international (who receive scholarships from the Hungarian government) students have health insurance (social security number, SSN). If students do not have SSN they can use private insurance such as Generali stadium health insurance, which must be paid by the student. As long as students have health insurance, the psychological services they receive are also free.

### Data collection and measurements

Online questionnaires were used to collect data. Students were requested to complete the questionnaire by clicking on the link given on the teaching platform before or after the teachers delivered the lecture or practice. The questionnaire took about 10–15 min to complete. The questionnaires were available in both English and Hungarian, and participants were required to fill in the informed consent before starting the online survey. Only fully completed questionnaires were taken into account. In our online questionnaire, respondents were required to answer all questions, as it was designed to prevent the submission of incomplete responses. This approach ensured that participants could only proceed with the survey after providing answers for all the questions.

#### Socio-demographic characteristics

Age: Students were asked to provide their age in years. For data analysis, age was divided into two categories: 18–25 and 26–37 years. Sex: Students were given the choice of choosing either a male or female sex on the questionnaire. Years of study: the years of study were categorized into ‘preclinical (1st/2nd)’ and ‘clinical (3rd/4th/5th/6th)’. Relationship status was dichotomized as ‘not in relationship (single/divorced/living separated)’ and ‘in relationship (married/common-law marriage/living together/ having a partner but not living together)’. The financial situation of the students’ family was evaluated by a 5-point Likert scale, which was dichotomized as ‘poor income’ (very bad/bad/average) and ‘high income (good/very good)’.

**Table 3:** Multivariable logistic regression analysis of visiting a GP

Characteristics		Total (688)			Hungarian (362)			International (326)	
	AOR	95% CI	p value	AOR	95% CI	p value	AOR	95% CI	p value
*Health behaviours*									
Alcohol consumption									
No	1			1			1		
Yes	1.26	0.89–1.79	0.191	1.07	0.63–1.80	0.804	1.37	0.84–2.23	0.204
Sexual intercourse									
No	1			1			1		
Yes	1.35	0.98–1.87	0.067	1.04	0.67–1.63	0.848	1.86	1.14–3.01	**0.012**
*General health status*									
Chronic illness									
No	1			1			1		
Yes	3.50	2.13–5.76	**< 0.001**	3.70	1.93–7.09	**< 0.001**	3.22	1.46–7.07	**0.004**
BMI									
≥ 25.0 kg/m^2^	1			1			1		
≤ 24.9 kg/m^2^	0.78	0.53–1.14	0.190	0.62	0.33–1.15	0.128	0.95	0.57–1.58	0.831
Nagelkerke R^2^		0.080			0.081			0.098	
Hosmer and Lemeshow Test		*P* = 0.577			*P* = 0.768			*P* = 0.257	

AOR = adjusted odds ratio, CI = confidence interval, BMI = body mass index

Adjusting for socio-demographic factors (age, sex, years of study, relationship status, economic status)

### Health behaviours

Smoking and alcohol consumption: if they smoked or drank alcohol, the response options were Yes (yes, occasionally/yes, regularly) or No. Sexual intercourse: if the students ever had sexual activity during medical school, and the response options were Yes or No.

### Health status

Chronic diseases: if medical students ever experienced any chronic illness during their study period, either self-determined or diagnosed by a doctor; response option was Yes or No. Body mass index (BMI), there were two categories, underweight and normal: BMI ≤ 24.9 kg/m^2^ and overweight and obese: BMI ≥ 25.0 kg/m^2^. Self-rated general health and mental health reported by using a five-point Likert scale (1 = very bad, 5 = very good). The respondents had to answer the question regarding general health, “how do you evaluate your general health status?“ and for mental health they were asked, “how do you evaluate your mental health status?“. For the purposes of data analyses, self-rated health (SRH) was categorized as good (scores 4 and 5) and poor (scores 1 to 3).

Perceived stress scale (PSS): The 10-item Perceived Stress Scale (PSS-10) [25] is a 10-item questionnaire originally developed by Cohen et al. (1983). Respondents were asked how often they felt a certain way on a five-point scale from ‘never’ to ‘very often’. PSS is not a diagnostic instrument, and the developer has not published any score cut-offs [26]. In the current study, for the purposes of data analysis, PSS was categorized as lower stress (score < 14) and higher stress (score ≥ 14) categories refer to previous study [27].

**Table 4:** Univariable logistic regression analysis of visiting a psychologist

Characteristics		Total (688)			Hungarian (362)			International (326)	
	OR	95% CI	p value	OR	95% CI	p value	OR	95% CI	p value
*Socio-demographic factors*									
Age (years)									
18–25	1			1			1		
26–37	1.30	0.75–2.26	0.339	1.39	0.62–1.11	0.418	1.41	0.65–3.04	0.376
Sex									
Male	1			1			1		
Female	2.10	1.35–3.29	**0.001**	1.87	1.02–3.43	**0.043**	2.19	1.12–4.28	**0.022**
Years of study									
Preclinical	1			1			1		
Clinical	1.46	0.98–2.19	0.058	2.02	1.19–3.42	**0.009**	0.74	0.36–1.50	0.412
Relationship status									
Not in a relationship	1			1			1		
In a relationship	1.23	0.82–1.84	0.309	1.15	0.68–1.92	0.598	1.05	0.52–2.15	0.875
Economic status									
Low income	1			1			1		
High income	0.93	0.61–1.40	0.730	1.07	0.63–1.80	0.787	0.89	0.44–1.79	0.755
*Health behaviours*									
Smoking									
No	1			1			1		
Yes	1.02	0.63–1.64	0.920	1.19	0.64–2.19	0.588	0.86	0.41–1.84	0.708
Alcohol consumption									
No	1			1			1		
Yes	1.73	1.08–2.79	**0.024**	1.97	0.96–4.05	0.061	1.35	0.70–2.63	0.364
Sexual intercourse									
No	1			1			1		
Yes	1.06	0.71–1.58	0.750	0.93	0.55–1.57	0.796	1.15	0.61–2.15	0.653
*Mental health status*									
PSS									
Lower	1			1			1		
Higher	2.21	1.43–3.43	**< 0.001**	2.17	1.24–3.79	**0.007**	2.11	1.03–4.33	**0.041**
Self-rated mental health									
Good	1			1			1		
Poor	3.12	2.01–4.83	**< 0.001**	2.94	1.67–5.18	**< 0.001**	3.37	1.68–6.77	**0.001**

OR = odds ratio, CI = confidence interval, BMI = body mass index

### Healthcare-seeking

Healthcare-seeking behaviour is defined as any activity performed by those who assumed they had a health issue or became ill with the intention of discovering an appropriate treatment [28]. Researchers determined that visits to the GP and psychologist are forms of healthcare-seeking behaviours. Visiting a GP or a psychologist: students answered the question if they visited the family doctor in the previous year, if they visited a psychologist in the previous year, and the response options were Yes and No.

### Statistical analysis

Data were analysed by IBM SPSS ‘Statistics 28.0’. Socio-demographic characteristics, health behaviours, health status and healthcare seeking were analysed using chi-square test. Univariable logistic regression analysis of these variables was performed to evaluate unadjusted relationships, only variables that have a p value < 0.05 were carried out by multivariable analysis. Multivariable logistic regression models were constructed to evaluate adjusted relationships, the adjusted variables were socio-demographic factors (age, sex, years of study, relationship status, and economic status). All analyses were carried out as a comparison of the Hungarian and international students. In the logistic regression analysis Nagelkerke R^2^ values were used to evaluate the explanatory power of the models, whereas Hosmer and Lemeshow proposed a goodness-of-fit test.

**Table 5:** Multivariable logistic regression analysis of visiting psychologist

Characteristics		Total (688)			Hungarian (362)			International (326)	
	AOR	95% CI	p value	AOR	95% CI	p value	AOR	95% CI	p value
*Socio-demographic factors*									
Sex									
Male	1			1			1		
Female	1.98	1.25–3.13	**0.004**	1.78	0.95–.3.35	0.072	2.21	1.11–4.41	**0.024**
Years of study									
Preclinical	1			1			1		
Clinical	1.29	0.85–1.95	0.228	1.74	1.00–3.01	**0.048**	0.70	0.34–1.47	0.352
*Health behaviours*									
Alcohol consumption									
No	1			1			1		
Yes	1.71	1.05–2.79	**0.032**	2.01	0.96–4.23	0.064	1.33	0.67–2.64	0.415
*Mental health status*									
PSS									
Lower	1			1			1		
Higher	2.70	1.68–4.33	**< 0.001**	1.28	0.68–2.41	0.440	1.28	0.59–2.76	0.530
Self-rated mental health									
Good	1			1			1		
Poor	1.30	0.80–2.11	0.284	2.45	1.31–4.60	**0.005**	3.08	1.47–6.45	**0.003**
Nagelkerke R^2^		0.109			0.118			0.108	
Hosmer and Lemeshow Test		0.644			0.102			0.800	

AOR = adjusted odds ratio, CI = confidence interval, BMI = body mass index

Adjusting for socio-demographic factors (age, sex, years of study, relationship status, economic status)

### Ethics

The study protocol was reviewed and approved by the Human Institutional and Regional Biomedical Research Ethics Committee, University of Szeged, Hungary (license number: 4936). All participants were informed of the objectives and procedures of the study and their rights to withdraw from the study. Informed consent was obtained from all included participants. Anonymous data were collected and held securely.

## Results

### Characteristics of the sample

The characteristics of the respondents are described in Table 1. The majority (86.6%) of the students were aged 18–25 years with the mean age of 22.47 ± 2.75 years. There were significant differences between the Hungarian and the international medical students in socio-demographic factors, such as age, sex, year of study, relationship and economic status.

There was no significant difference in smoking behaviour between Hungarian (21.3%) and international students (23.9%) (p = 0.405), while alcohol consumption and sexual activity differed significantly between the two groups, p < 0.001 and p = 0.003, respectively. Hungarian (alcohol 77.9%; sexual intercourse 58.3%); International (alcohol 61.3%; sexual intercourse 46.9%).

There was no significant difference in self-rated health and chronic illnesses between the two groups. Meanwhile, more than a quarter (29.8%) of the international students reported having a BMI ≥ 25.0 kg/m^2^, whereas 16.0% of the Hungarian students had a BMI ≥ 25.0 kg/m^2^ (a statistically significant difference; p < 0.001).

In the total sample, there was a difference in the proportion of visits to the GP (56.8%) and the psychologist (17.2%); the number of visits to the psychologist was lower. Concerning the number of visits to the GP between Hungarian (57.7%) and international (55.8%) students, there was no significant difference. On the other hand, there was a statistically significant difference between the two groups regarding visits to the psychologist: Hungarian (19.9%) and international (14.1%) (p = 0.045). This difference could potentially be attributed to the availability of psychological services, which might vary between the groups and influence the utilization of such services.

### Medical students visit to the GP

In the univariable logistic regression (Table 2), there was no significant relationship identified between the socio-demographic characteristics and the GP visits by medical students in both groups.

Health behaviours significantly associated with visiting the GP were found to be alcohol consumption and sexual intercourse in the total sample. While smoking behaviour had no significant relationship in either group. General health status significantly associated with the GP visits included the presence of a chronic disease as well as BMI scale in the total sample. In contrast, self-rated general health had no significant association in either group.

International students were more likely to visit GP when they had chronic illness and experienced health behaviour (such as alcohol and sexual intercourse). Hungarian students were more likely to visit GP when they had chronic illness. In the total sample, alcohol, sexual intercourse, chronic illness and BMI associated with visiting GP among medical students.

According to the multivariable logistic regression (Table 3), only chronic diseases were significantly associated in the total sample. International medical students were more likely to visit a GP when they had experienced sexual intercourse (AOR = 1.86, 95%CI 1.14–3.01, p = 0.012) and had a chronic disease (AOR = 3.22, 95%CI 1.46–7.07, p = 0.004). On the other hand, Hungarian medical students were more likely to visit a GP when they had chronic disease (AOR = 3.70, 95%CI 1.93–7.09, p < 0.001).

### Medical students visit to the psychologist

According to the univariable logistic regression analysis (Table 4), socio-demographic factors that have a significant relationship with visits to a psychologist was sex in both groups, but the year of study was relevant only in the Hungarian group. Health behaviours included alcohol consumption were significantly related to visits to the psychologists in the total sample. Moreover, mental health status, PSS and self-rated mental health had a significant relationship in all groups.

Table 4 indicates that among international students, a higher perception of stress and poor self-rated mental health were associated with a greater likelihood of visiting a psychologist. On the other hand, within the group of Hungarian students, visiting a GP was more probable when they were in clinical years, experienced higher levels of perceived stress, and reported poorer mental health.

With regard to the multivariable analysis (Table 5), there was a significant correlation between gender, alcohol consumption, and PSS in the total sample of the visits to the psychologists. Hungarian medical students were more likely to visit the psychologists when they were in the clinical years (AOR = 1.74, 95%CI 1.00-3.01, p = 0.048) and had poor self-rated mental health (AOR = 2.45, 95%CI 1.31–4.60, p = 0.005). While female international (AOR = 2.21, 95%CI 1.11–4.41, p = 0.024) students and those who had poor self-rated mental health were more likely to seek psychological help (AOR = 3.08, 95%CI 1.47–4.45, p = 0.003).

## Discussion

### Main findings

The findings revealed that medical students’ utilization of medical assistance from GPs or psychologists remained low, aligning with similar observations made in other studies [1, 4]. Medical students may feel they have the knowledge to overcome their health problems by seeking help from friends or family members [3, 20]. The same opinion has been expressed in a study that students tend to avoid or delay disclosure, and they seek help because of the perceived risk to their future [6, 16]. Meanwhile, in another study, it has been suggested that the reason for coming to professional healthcare was not because it would hinder the students’ studies or reduce their achievement because of the disease they were having [4], but they were reluctant to come to the GP for reasons of the type and level of the disease they were experiencing [20, 29].

Our study showed that both Hungarian and international medical students who had chronic illnesses had the possibility to visit the GP. This finding was supported by a study which stated that students had symptoms of chronic diseases, such as respiratory, gastrointestinal, musculoskeletal symptoms or miscellaneous would come to visit the GP [29]. The same thing has also been described by previous studies which stated that health seeking behaviour among medical students might be influenced by the presence or absence of chronic diseases the students had [30]. However, another study claims that only students with symptoms of the disease at a severe stage would come to seek medical assistance [7, 31]. When analysing between local and international students, there may be several things that need to be considered, as it is known in the previous research that the mental health of international students might be influenced by the process of acculturation that they experience [11].

Likewise, students who often engage in risky health behaviours would have an impact on their physical and mental health, so that these students need appropriate health assistance [32]. The current findings suggest that international medical students who were sexually active were more likely to visit a GP. Hobs reports in his research that family doctors are the most common providers of support (47.5–54.8%), less than half of the individuals experiencing unpleasant sexual difficulties sought help or advice to health professionals [33]. The primary obstacle to students seeking medical care (70.4%) and students in need of sexual health counselling (72.2%) was acceptance of services [34].

The findings of the current study show that students were less likely to come to the psychologist than to the GP. This is of particular concern even though 50.9% of the students reported experiencing poor mental health, they were reluctant to come for psychological help. This might be influenced by the stigmatization of students when they have poor mental health problems [20, 35]. Worries about confidentiality were only seen as a barrier to seeking help for mental health problems [20] to consult friends and⁄ family informally about symptoms relating to mental health problems [4].

A prior study has revealed that men and women exhibit comparable help-seeking behaviours [36]. However, in the current study, it was observed that female students were more likely to seek the advice of a psychologist, and this finding was also present in the study of Mou, that is, women were more likely than men to take precautions [37, 38]. The likelihood of contracting a disease, how well people respond to treatments, and how frequently they seek medical attention have all been found to be influenced by sex and social circumstances [39]. Another study has claimed that patients’ self-reports revealed gender variations in the way they sought medical attention with women saying they contacted their primary care physician more frequently than males for both physical and mental health issues [38, 40].

The study conducted by Sawaha highlights that the academic year affects the search for health assistance [30] similarly to the findings in the current study where clinical students tried to seek help for psychological problems more often by consulting a psychologist. Students in higher academic years had a higher probability to be in the risk pattern of burnout, so they needed to come to the psychologist more often [24]. In addition, this study found a link between psychological visits and perceived stress, and prior research indicated that medical students generally experienced greater levels of perceived stress and emotional distress [24]. A more thorough understanding of how various sorts of stressors affect college students’ mental health will certainly allow any such efforts to more accurately identify and offer options to those who need support [41].

The recent findings argue that alcohol consumption was associated with seeking help from a psychologist. Consequently, drinking issues may have a very negative effect on mental health. One recent study showed that drinking alcohol were more likely to have mental health problems [23]. Excessive alcohol consumption and alcoholism can exacerbate pre-existing disorders like depression, or they may lead to the development of new issues, such as anxiety, depression or significant memory loss [14, 42].

### Strengths and limitations

In this study, health issues concerning both general health and mental health were discussed by using self-rated health assessments that are easy to assess by respondents and do not require much time in answering questionnaires. As another strength of the survey, a perceived stress scale that is easily understood by respondents in assessing the perception of stress was used. Nevertheless, despite the use of valid measurements and online survey measure, this study has several limitations. First, the cross-sectional study design may cause result bias. Second, convenience sampling was employed to recruit medical students’ participation, which may affect the representativeness of our findings. Third, since the data collection was conducted during the COVID-19 pandemic in Hungary, it may have an impact on health status (including mental health) and healthcare-seeking behaviours of students. Fourth, data on the students’ country of origin and the use of secondary care were not available or were not analysed in this study.

Future studies should consider using longitudinal study design, mixed method (qualitative and quantitative), dig deeper into the reasons why students seek medical assistance other than the variables in this study. The reasons why students visit psychology less frequently than to the GP can be explored by in-depth interviews. Future researchers can explore the barriers to seeking help among medical students.

## Conclusion

Healthcare-seeking was influenced by socio-demographic characteristics, health behaviour and health issues among medical students. This finding is encouraging medical schools to promote students to ask for assistance and come forward with problems early as well as increase student awareness in order to reduce risky behaviours. Therefore, in addition to their patients, a medical student’s future career depends on the ability to fully comprehend healthcare attitudes and to develop solutions that will improve health behaviour and to get appropriate health care.

## Data Availability

The datasets used during the current study are available from the corresponding author on reasonable request.

## Abbreviations

GP: General practitioner
OHI: Online health information
USZ: University of Szeged
SRH: Self-rated health
PSS: Perceived stress scale
BMI: Body mass index
OR: Odd Ratio
CI: Confidence Interval
