# Correction to: Healthcare-seeking of medical students: the effect of socio-demographic factors, health behaviour and health status – a cross-sectional study in Hungary

**DOI:** 10.1186/s12889-024-18928-6

**Published:** 2024-05-27

**Authors:** Afriza Umami, Viktória Zsiros, Ágnes Maróti-Nagy, Zsuzsanna Máté, Sudalhar Sudalhar, Regina Molnár, Edit Paulik

**Affiliations:** 1 Department of Public Health, Albert Szent-Györgyi Medical School, University of Szeged, Dóm tér 10, Szeged, H-6720 Hungary; 2 Stikes Muhammadiyah Bojonegoro, Bojonegoro, Indonesia

**Correction to**: ***BMC Public Health* 23, 2126 (2023)**.

10.1186/s12889-023-17041-4

The original publication of this article contained an error in connection to the BMI. This caused an error in:

- The 3rd paragraph of the results section.
- Tables 1, 2 and 3.

The incorrect and correct information are shown in this correction article. The original article has been updated.

## Incorrect

**Table 1**

**Table:** 

BMI							
≤ 24.9 kg/m^2^	155	22.5	58	16.0	97	29.8	< 0.001
≥ 25.0 kg/m^2^	533	77.5	304	84.0	229	70.2	

**Table 2**

**Table:** 

BMI									
≤ 24.9 kg/m^2^			1			1			1
≥ 25.0 kg/m^2^	0.69	0.47–0.99	**0.045**	0.56	0.31–1.03	0.061	0.75	0.46–1.21	0.248

**Table 3**

**Table:** 

BMI									
≤ 24.9 kg/m^2^	1			1			1		
≥ 25.0 kg/m^2^	0.78	0.53–1.14	0.190	0.62	0.33–1.15	0.128	0.95	0.57–1.58	0.831

### Results section

Meanwhile, more than a quarter (70.2%) of the international students reported having a BMI ≥ 25.0 kg/m2, whereas 84.0% of the Hungarian students had a BMI ≥ 25.0 kg/m2 (a statistically significant difference; *p* < 0.001).

## Correct

**Table 1**

**Table:** 

BMI							
≥ 25.0 kg/m^2^	155	22.5	58	16.0	97	29.8	< 0.001
≤ 24.9 kg/m^2^	533	77.5	304	84.0	229	70.2	

**Table 2**

**Table:** Table 2

BMI									
≥ 25.0 kg/m^2^			**1**			1			1
≤ 24.9 kg/m^2^	0.69	0.47–0.99	**0.045**	0.56	0.31–1.03	0.061	0.75	0.46–1.21	0.248

**Table 3**

**Table:** 

BMI									
≥ 25.0 kg/m^2^	1			1			1		0.831
≤ 24.9 kg/m^2^	0.78	0.53–1.14	0.190	0.62	0.33–1.15	0.128	0.95	0.57–1.58	0.831

### Results section

Meanwhile, more than a quarter (29.8%) of the international students reported having a BMI ≥ 25.0 kg/m^2^, whereas 16.0% of the Hungarian students has a BMI ≥ 25.0 kg/m^2^ (a statistically significant difference; *p* < 0.001).

